# BRENDA in 2015: exciting developments in its 25th year of existence

**DOI:** 10.1093/nar/gku1068

**Published:** 2014-11-05

**Authors:** Antje Chang, Ida Schomburg, Sandra Placzek, Lisa Jeske, Marcus Ulbrich, Mei Xiao, Christoph W. Sensen, Dietmar Schomburg

**Affiliations:** 1 Department of Bioinformatics and Biochemistry, Technische Universität Braunschweig, Langer Kamp 19 B, D-38106 Braunschweig, Germany; 2 The Jackson Laboratory, 263 Farmington Avenue, Farmington, CT 06030, USA; 3 Department of Biochemistry & Molecular Biology, Faculty of Medicine, University of Calgary, 3330 Hospital Drive N.W., Calgary, Alberta T2N 4N1, Canada

## Abstract

The BRENDA enzyme information system (http://www.brenda-enzymes.org/) has developed into an elaborate system of enzyme and enzyme-ligand information obtained from different sources, combined with flexible query systems and evaluation tools. The information is obtained by manual extraction from primary literature, text and data mining, data integration, and prediction algorithms. Approximately 300 million data include enzyme function and molecular data from more than 30 000 organisms. The manually derived core contains 3 million data from 77 000 enzymes annotated from 135 000 literature references. Each entry is connected to the literature reference and the source organism. They are complemented by information on occurrence, enzyme/disease relationships from text mining, sequences and 3D structures from other databases, and predicted enzyme location and genome annotation. Functional and structural data of more than 190 000 enzyme ligands are stored in BRENDA. New features improving the functionality and analysis tools were implemented. The human anatomy atlas CAVEman is linked to the BRENDA Tissue Ontology terms providing a connection between anatomical and functional enzyme data. Word Maps for enzymes obtained from PubMed abstracts highlight application and scientific relevance of enzymes. The EnzymeDetector genome annotation tool and the reaction database BKM-react including reactions from BRENDA, KEGG and MetaCyc were improved. The website was redesigned providing new query options.

## INTRODUCTION

BRENDA (BRaunschweig ENzyme DAtabase; http://www.brenda-enzymes.org/), the major public information system for functional and molecular properties of enzymes, celebrated its 25th anniversary in 2013. Originally it was started as a series of books [Handbook of Enzymes, (1)] to provide a substantial enzyme repository, based on the Enzyme Commission (EC) classification system of the IUBMB [International Union of Biochemistry and Molecular Biology, (2)]. Since 1998 BRENDA has been available via the Internet with a first query system. With the fast growing number of data and knowledge in the ‘OMICS’ area, the continuous integration of new data and new developments in BRENDA has to meet the expectations as an enzyme and enzyme function data resource for systems biology, biotechnology, medical research and related fields. BRENDA contains information on all classified enzymes of organisms of all taxonomic groups. The information encompasses data on the catalyzed reaction, enzyme–ligand interaction, inhibition, occurrence, stability, kinetics, mutants, application, protein sequence and structure, disease-related data, etc. The manually curated BRENDA core contains enzyme-specific data, extracted by experts in biochemistry, biology and chemistry. Each single entry is linked to a reference, the enzyme source, the tissue, the subcellular localization and, if available, to a protein sequence.

Furthermore, BRENDA includes large amounts of information on the occurrence of enzymes in organisms, their cellular localization, their involvement in human diseases, their active centers and interfaces, as well as additional kinetic data, resulting from text mining and analysis methods and bioinformatics approaches (3,4).

The enzyme data stored in BRENDA include textual, single numeric and numeric range data, diagrams and other graphic data, one-, two- and three-dimensional structural data. The query system offers an easy access to the complex data stored for >60 data categories. From simple ‘Google-like’ queries performed via the ‘Quick Search’ option to more elaborate queries (‘Advanced Search’, ‘Fulltext Search’) and the ‘Substructure Search’ by a structure editor BRENDA provides a user-friendly system for retrieving the enzyme-specific information.

The ‘BRENDA Ligands’ database is an essential part of the repository. The term ‘ligands’ covers all compounds interacting with enzymes, i.e. substrates and products, inhibitors, cofactors, activating substances, etc. 193 000 small molecules or other ligands are stored as structures, names or synonyms, and can be searched and displayed. A graphical substructure search is included.

In 2003, BRENDA started to develop the BRENDA Tissue Ontology (BTO), a structured comprehensive Encyclopedia of tissue terms from multicellular organisms (5). The BTO allows direct access to information about enzymes, isolated from or detected in specific body parts, organs, tissue, cell types and cell lines.

Additionally, the BRENDA portal offers access to further enzyme-related data, i.e. the ‘Genome Explorer’ to display and compare enzymes of a complete genome, the ‘TaxTree Explorer’ showing all organisms of the NCBI taxonomy database (6) including links to BRENDA enzyme entries, the ‘EC Explorer’, displaying the hierarchical EC classification system with direct access to the BRENDA data, and the ‘Ontology Explorer’, comprising various ontologies, connected to BRENDA enzyme entries, as well.

Since the last publication in 2013 new features and improvements are implemented in BRENDA. The EnzymeDetector (7) and BKM-react (8) are substantially upgraded. Newly integrated are ‘Word Maps’ that provide graphical information on terms and perceptions associated with specific enzymes. The human anatomical atlas, which was derived from ‘Terminologia Anatomica’ (9) and originally used in the CAVEman project (10), was integrated into BRENDA and linked to the enzyme data (11). Following an extended user survey, the BRENDA website was redesigned to facilitate mobile access and to provide a modern design (but still providing the user with the option to stay with the classical search layout).

## CONTENTS

The classical core of the database is composed of manually extracted literature data. Literature references published between 1939 and today are annotated. They provide data for 6547 enzyme classes (EC numbers). Of these, 5374 EC classes represent the official EC numbers approved by the IUBMB and derived from the ExplorEnz database (12). Each year ∼300 new EC classes are issued by the IUBMB, with strong involvement of BRENDA scientists. These are integrated into BRENDA as soon as they are officially approved.

Four-hundred nineteen EC classes are preliminary BRENDA-supplied EC numbers. Most of these enzymes are either waiting to be approved by the enzyme commission of the IUBMB or are lacking some of the details required for a classification by the enzyme commission. In many cases, the substrate specificity or the cofactor requirements are not sufficiently analyzed. In order to receive an EC number, experimental evidence for the catalyzed reaction is indispensable. The remaining 754 EC classes belong to transferred or deleted entries. In the course of the continuous enzyme classification process, sometimes enzymes are relocalized by the enzyme taskforce to different subgroups taking into account that the specificity is found to be different than originally reported.

The enzymes are characterized with up to 60 attributes. The minimum requirement is an EC number, an organism name and a literature citation. The main categories cover the catalyzed reactions and specificity, kinetic data, the protein structure, the activity range and the stability. Where available the kinetic values under which the respective values were obtained are given with the experimental conditions thus providing comparability. The manually annotated part of BRENDA amounts to 3 million data obtained from 135 000 references for 11 000 different organisms. Table 1 shows the amount of data in selected data categories.

**Table 1.** BRENDA: the number of entries in selected data fields

Enzyme information	Entries
Substrate and products	381 855
Inhibitors	183 745
Cofactors	13 696
Metal and ions	33 990
Activating compounds	25 735
*K*_M_-values	127 882
*K*_i_-values	35 730
*k*_cat_-values	56 613
Specific activity	44 141
IC_50_	44 229
Localization and source/tissue	91 767
Enzyme names and synonyms	95 595
Citations (manually annotated)	136 937
Isolation and preparation/crystallization	64 324
Enzyme structure	65 434
Mutant enzymes	68 238
Stability	45 628
Enzyme application	14 000

The numbers refer to the combination of enzyme protein, source organism and literature reference. The term enzyme protein refers either to a protein sequence or to a protein isolated from a given organism without its sequence having been determined.

In order to present the data in a uniform manner, BRENDA is based on ontologies and controlled vocabularies as far as possible. The organism names are based on the NCBI Taxonomy. The occurrence in tissues and organs is linked to the BTO (see below). Subcellular localization follows the nomenclature of the Gene Ontology (13). The ∼193 000 molecules that serve as substrates, products, cofactors, activators or inhibitors—referred to as ‘ligand’—prove to be a challenge since synonyms must be clearly labeled. Essential information on the individual compounds is displayed in the ‘ligand view’.

The manually annotated and curated data are completed by a wealth of semi-automatically or automatically retrieved data.

FRENDA (Full Reference ENzyme DAta) and AMENDA (Automatic Mining of ENzyme DAta) contain data on the occurrence of enzymes in organisms, tissues or expressed in cell cultures retrieved by text mining of titles and abstracts of the NCBI PubMed database (14). These two repositories contain data for ∼30 000 organisms, of which one-third also occur in the manually annotated core database. While FRENDA lists all co-occurrences of an enzyme associated with an organism, AMENDA is based on more elaborate text mining strategies verification steps. The accuracy and precision of the text mining results have been highly increased, and false-positive entries were removed. Two further validation steps were integrated to remove wrong associations between ‘Source Tissue’ and ‘Organism’ entries (e.g. leaf–*Homo sapiens*) and ‘Intracellular localization’ and ‘Organism’ entries (e.g. mitochondria–*Escherichia coli*), respectively. Furthermore incorrect organism names in the dictionaries were removed.

The role of enzymes in disease development, diagnosis or treatment is of extremely high relevance. A combination of text mining and algorithm-based classification approaches (DRENDA, Disease RElated ENzyme information DAtabase) allowed the identification of more than 600 000 articles describing causal interactions between enzyme function of malfunction and diseases (e.g. 180 000 papers on infection) (15).

KENDA (Kinetic ENzyme DAta) is a database containing kinetic enzyme data, extracted from PubMed literature abstracts via text mining methods. The procedure makes use of sets of kinetic terms and their modifications, words for the interpretation of sentences, names for metabolites and ligands from BRENDA and PubChem, enzyme names and organism from the PubMed taxonomy.

The compilation of these databases is based on the ontologies and controlled vocabularies in BRENDA, on the BRENDA ligand names, the PubChem (16) ligand names, the NCBI Taxonomy and the MESH terms, followed by a support-vector machine classification step. Table 2 gives an overview on the content of the various text mining databases assembled in the BRENDA portal.

**Table 2.** BRENDA data retrieved by text mining processes

FRENDA	
Reference-organism	7 583 318
AMENDA	
Reference-organism	4 034 106
Reference-organism-source tissue	1 127 159
Reference-organism-subcelluar localization	232 084
DRENDA	
Diseases connected to enzymes	135 508
References for enzyme-connected diseases	1 130 391
KENDA	
Literature abstracts with enzyme kinetic data	7832

Links to other databases e.g. KEGG (17), MetaCyc (18), ExplorEnz, UniProt (19), PDB (20), NCBI are included, where possible.

An elaborate set of search modes, analysis tools and data retrieval options facilitates the access. New developments are described below.

## NEW DEVELOPMENTS AND MAJOR IMPROVEMENTS

### Word maps

Word maps have become widely used in many fields of the natural and social sciences as well as in everyday life to illustrate association of terms and concepts. The more frequently a word is associated with an enzyme in a text and the higher its specificity is, the more prominent it will be presented in the graphical representation of the word map. Other features like color or links are used to provide e.g. information on the category of the displayed term. Word maps provide a new option to quickly obtain information on the perceptions scientific authors have when they publish facts on enzymes. Word maps are now accessible from the BRENDA result pages. The terms are classified with respect to organism, source tissue, localization, ligand, disease and application, which determines the coloring in the final diagram.

**Figure 1.**
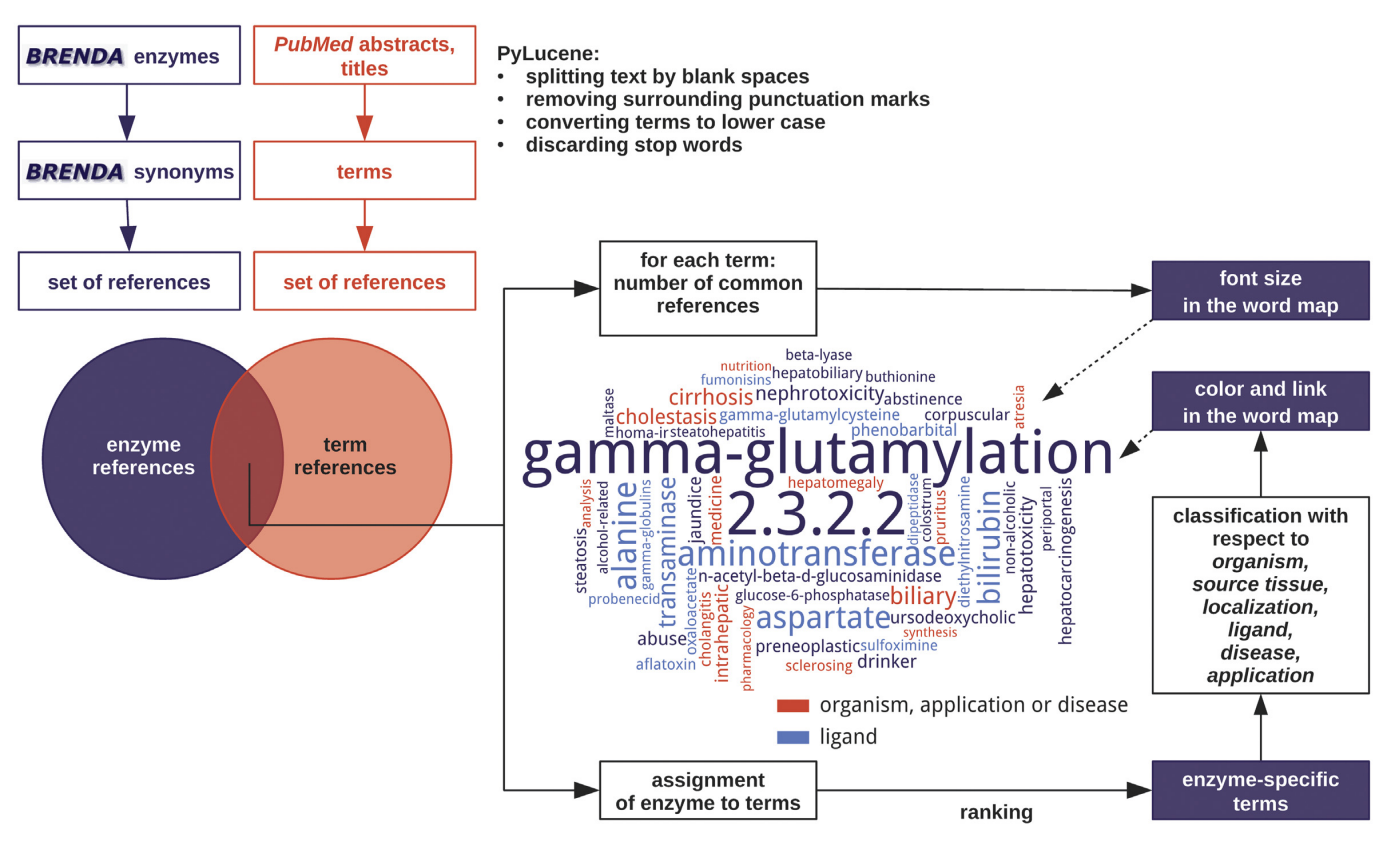
The workflow for the construction of enzyme word maps illustrated for γ-glutamyltransferase (EC 2.3.2.2).

In a multi-step process, terms that occur in PubMed titles or abstracts of papers on a certain enzyme are extracted and ranked with respect to their relevance. All extracted terms pass through 1. processing, 2. filtering and 3. ranking steps. Only a selected fraction is displayed in the word map. A detailed description of the work flow is shown in Figure 1. The Supplementary material contains a detailed description of the whole procedure.

The processing of the abstracts into single words and processing of the words (stemming, removal of non-characters, etc.) is done with ‘PyLucene’ (http://lucene.apache.org/pylucene/). This procedure results in a list of terms linked to the corresponding enzymes and references. Popular high-frequently occurring words are discarded. In order to achieve a meaningful representation, several evaluation steps are performed. For example non-enzyme-specific terms and very rare terms are removed.

### EnzymeDetector

In 2013, the EnzymeDetector was introduced in BRENDA. This platform provides a fast and comprehensive overview of the available functional predictions for enzymes encoded in bacterial and archaeal genomes. Enzyme functions from the main annotation databases are supplemented with our own functional predictions and experimental information from BRENDA and are subsequently integrated and evaluated. The user can customize the integrated weighing scheme and cutoffs of the different prediction methods and thus correct mis-annotations that may occur in one of the databases.

For the current BRENDA release the EnzymeDetector algorithm underwent a profound update.

- Bacterial strain denominations from the BRENDA manual annotation and AMENDA results increase the reliability of the prediction. Data that are only available for the genus level are now integrated with the respective lower rating.
- Orthology data of KEGG and annotations of PATRIC (21) were included as additional data sources.
- The database mapping between all integrated sources is now based on the locus-tags of the genes.
- The pre-computed BLAST-Matrix SIMAP (SImilarity MAtrix of Proteins) (22) was integrated into our own Basic Local Alignment Search Tool (BLAST) annotation (23). Those proteins that could not be found in SIMAP were aligned by proteinBLAST (version 2.2.29+). E-values, BLAST-Scores, identity and coverage of query sequences were compared to the complete SwissProt sequence set and stored to validate overall annotation data.
- In addition to the described sequence similarity-based annotations, predictions based on sequence patterns using the 2014 updated BREPS pattern tool (24) were integrated.
- Further recommendation for decision about correct gene annotations is supplied by the integration of PFAM-HMMs, a large collection of protein families, each represented by multiple sequence alignments and hidden Markov models (HMMs) (25,26).
- The algorithm was modified for the detection of bi-functional and multifunctional enzymes from the various annotation sources.

The current release (August 2014) of the EnzymeDetector contains processed data of 5244 chromosomes and plasmids. The annotations are mapped to 8.6 million genes including ∼230 archaeal genomes.

**Figure 2.**
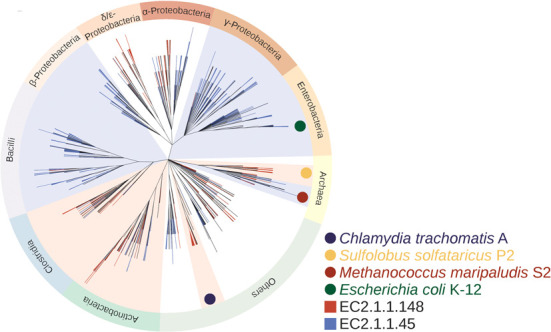
The distribution of thymidylate synthases (EC 2.1.1.45 and EC 2.1.1.148) in bacteria and archaea obtained from the EnzymeDetector, mapped on the NCBI taxonomic tree. Only thymidylate synthase annotations with a relevance score above 8 were included. The relevance score reflects the sum of the weights of the different annotation sources. In this scheme, manual annotations (e.g. BRENDA) get high rates, whereas predictions get low ones. For a detailed description see (7).

The newly designed EnzymeDetector website offers various ways to explore enzyme diversity within the database. The enzyme repertoire of whole phylogenetic clades can be analyzed (27,28). Figure 2 shows the distribution of the two thymidylate synthases (EC 2.1.1.45 and EC 2.1.1.148) in bacteria and archaea as obtained from EnzymeDetector, mapped on the taxonomic tree of the NCBI. These enzymes catalyze an essential step in the synthesis of pyrimidine deoxyribonucleotides. Several alternate pathways are observed which differ in the usage of the two key enzymes. β- and γ-proteobacteria, enterobacteria and bacilli are restricted to the use of EC 2.1.1.45. The classes *Actinobacteria*, *Chlamydia* and *Clostridia* predominately express EC 2.1.1.148, with tightly bound flavin adenine dinucleotide (FAD). *Archaea*, which are known for their metabolic versatility, are clearly split into two groups, using either the classical branch or displaying the alternate path with EC 2.1.1.148.

**Figure 3.**
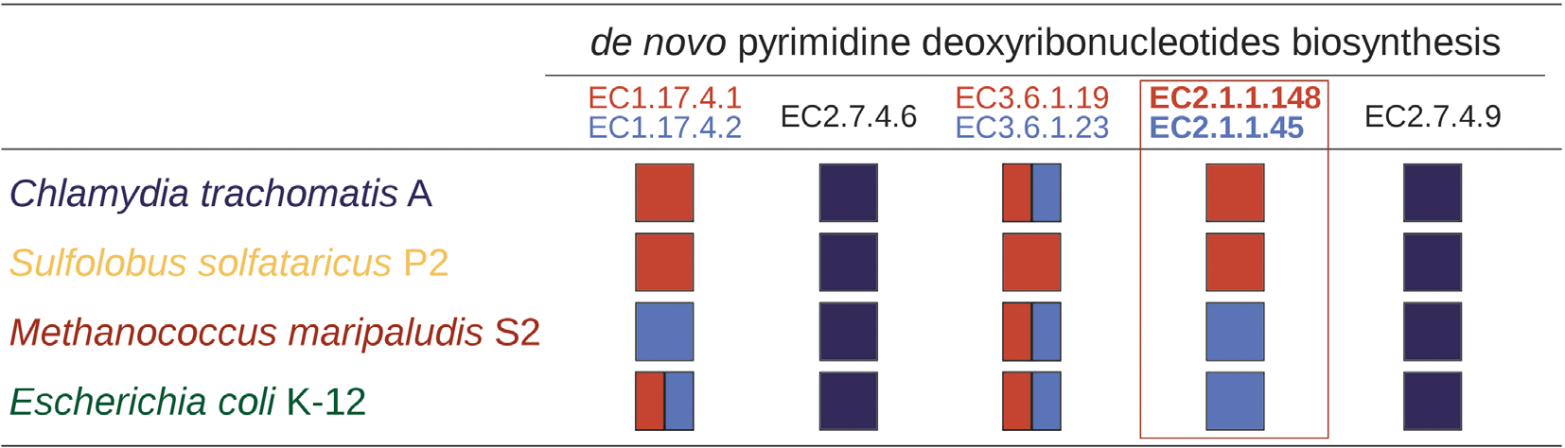
Enzymes in the pathway for the synthesis of pyrimidine deoxyribonucleotides detected by the EnzymeDetector, showing alternate routes. Red and light blue squares and rectangles represent alternate enzymes. Enzymes common to all pathway variants are shown in dark blue. The columns from left to right represent subsequent reaction steps.

EnzymeDetector is well suited to detect the presence or absence of all enzymes in a pathway for a selected organism. Hence it is possible to detect gaps or mis-annotations. Figure 3 shows all enzymes that participate in pathway variants of the synthesis of pyrimidine deoxyribonucleotides for *Chlamydia trachomatis*, *Sulfolobus solfataricus*, *Methanococcus maripaludis* and *E. coli* termed PWY-7184, PWY-7187, PWY-6545 and PWY-7198 in MetaCyc, respectively. In these pathways the organisms differ in the presence of EC 1.17.4.1/2, EC 3.6.1.19/23, and the two key enzymes for thymidylate synthesis (EC 2.1.1.45/148). Furthermore, it can be seen that for *M. maripaludis* EC 1.17.4.1 is not annotated. Instead this organism uses EC 1.17.4.2 for this reductive step. *S. solfataricus* known for its enzymes with broad substrate specificity only expresses EC 3.6.1.19 and does not depend on EC 3.6.1.23 which is specific for the hydrolysis of dUTP. A new EnzymeDetector website with a modern more functional web design has been launched. It is now integrated into the BRENDA website or accessible via http://edbs.tu-bs.de/. The weights given to different annotation sources can be adjusted and thus the output changes according to the chosen parameters. The results of the prediction can be checked by the statistical analysis included in the EnzymeDetector website. Filters allow the user to configure the display of information on the screen at will. The combination of the weighted integration of different annotation tools and sources, calculation of overall statistics, pathway coverage overview and comparisons between organisms makes EnzymeDetector a highly potent enzyme prediction tool, ideally suited for e.g. creation of metabolic models or analysis of metabolic pathway distribution between organisms. All results can be downloaded as spreadsheet, pdf or csv.

### Human anatomy atlas CAVEman/BTO

Biological databases have to be based on an accepted terminology to enable and facilitate the search for data. Thus enzymes, their sources and occurrence are described with controlled vocabularies, defining ‘recommended’ terms and their respective synonyms. In particular the occurrence of enzymes is embedded in numerous ontologies.

For human enzymes, a newly developed more detailed ontology for body parts, organs and tissues has been integrated. It is part of the software system CAVEman which was developed at the University of Calgary. CAVEman represents a three-dimensional digital atlas of the adult male human anatomy and is structured according to the nomenclature of ‘Terminologia Anatomica’. In order to integrate these terms into BRENDA, the XML-based ontology was converted into OBO format. In a next step, the terminology of CAVEman and the BTO were matched. Subsequently, the matches were checked manually and the terminology was changed in some areas in order to obtain the largest possible overlap. In this manner, 426 terms yielded a complete and 3272 a partial concordance, respectively. Of the partial matches 2700 terms could be assigned manually.

**Figure 4.**
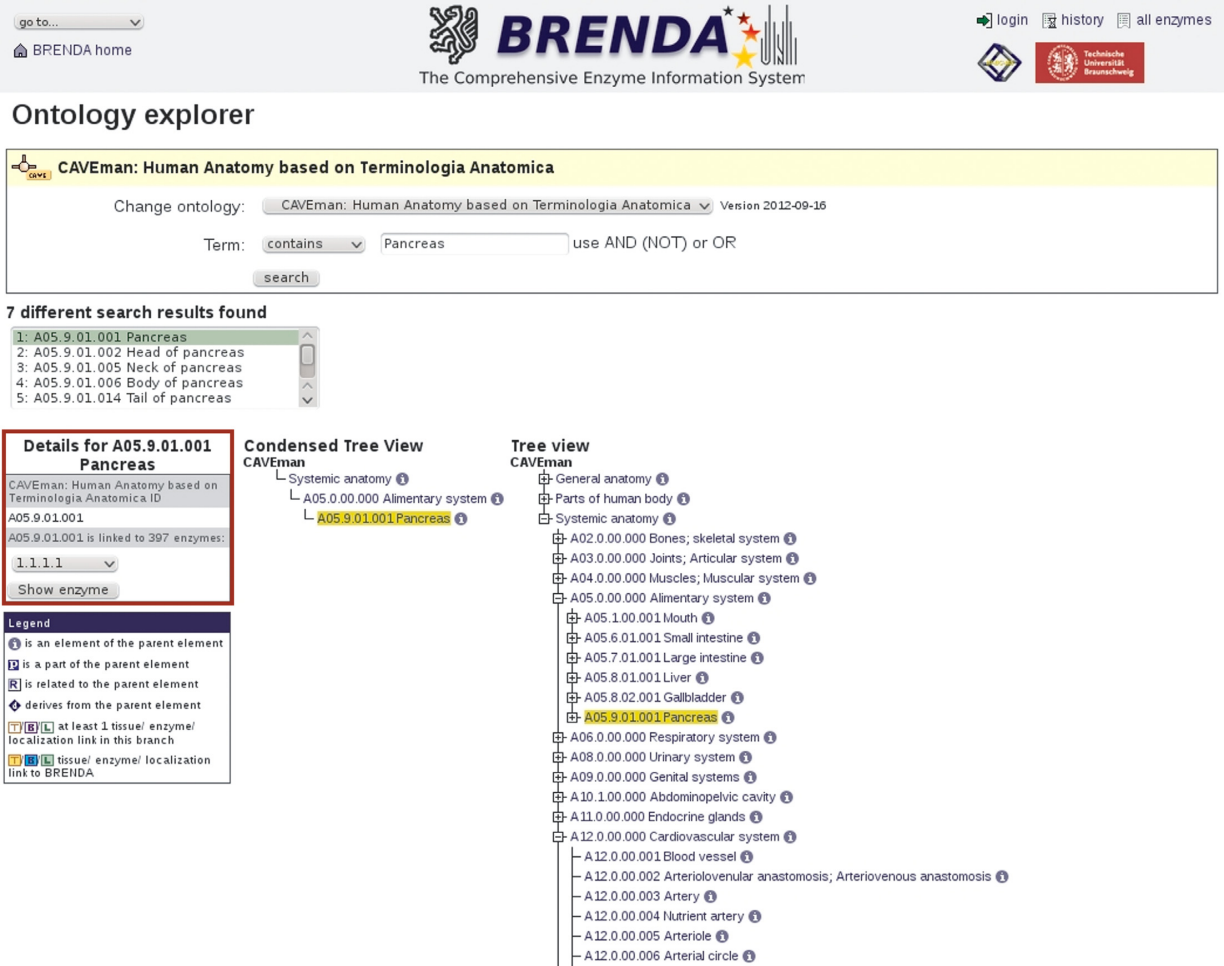
CAVEman human anatomy linked to enzyme data.

The CAVEman ontology was then inserted into the BRENDA Ontology Explorer. The terms are linked to the enzyme data. For example the term ‘pancreas’ is linked to 397 enzyme classes (see Figure 4). These links provide detailed information on the enzymes, their specificities and properties and their medical relevance. The combination of both anatomy and functional enzyme data, respectively, allows new insight into biomedical aspects of the human body in terms of disease-related gene expression or biochemical impact.

### BKM-react

The construction of cellular models includes intermolecular signal transduction, gene regulatory and metabolic submodels. For a realistic model of the cellular metabolism, a complete list of all enzyme-catalyzed reactions is an essential precondition. A small number of databases providing information on biochemical reactions exist, BRENDA, KEGG and MetaCyc being the most comprehensive. None of these databases offers a complete set of reactions. A combination of these three databases however allows a distinctly more realistic metabolic model than the use of a single one of these databases. On the other hand, it is essential that reactions do not occur several times in a model e.g. with different substrate names.

Since 2011 the BRENDA website gives access to BKM-react (Biochemical Reactions Aligned), an integrated and non-redundant biochemical reactions database. Since the publication of the original paper the number or reactions increased by about 20%. It contains now 21 665 unique enzyme-catalyzed reactions on natural substrates i.e. twice as much as any of the three integrated databases. Out of the 67 000 full reactions stored in BRENDA only those were included where the reactions were described in the paper to occur in a certain organism.

The website now has a new layout with the main focus on a substrate/product search including all synonyms. For example, the reactant query ‘*(6R)-*tetrahydrobiopterin’ gives one reaction without the search for synonyms and 25 reactions with the synonym search.

In addition to the main search field, the user can utilize the search fields Reaction Partner, Recommended Name, EC Number, KEGG Pathway Name, MetaCyc Pathway Name, Reaction ID BRENDA, Reaction ID KEGG, Reaction ID MetaCyc, Stoichiometry Check, Missing Substrate and Missing Product. Also a full text search using simple BOOLEAN operations with the terms ‘AND’, ‘OR’ ‘AND NOT’ is integrated.

### New BRENDA website

The BRENDA website has been completely modified and now presents a new and modern easy-to-use interface. The new design is based on user surveys and user feedback and is optimized for quick access of the enzyme information. The most obvious changes are the simplified search options. For users unfamiliar with the database contents, a fulltext search is included as the default query option which means that for a quick database search he/she is not obliged to get acquainted with the various data fields and search options. The result of this query is a table showing the number of times the query term occurs in different data fields. Details are then obtained by selection of the appropriate data field. Experienced users can continue to search via the data field-specific classic query options. The former navigation panel on the left side is replaced by a drop down list in the top of each page. Frames are no longer used and thus more browsers are supported.

More specific, advanced, graphical-input-supported or graph-based queries such as the ligand substructure search, sequence search, EC-tree search, organism-tree search or ontology search are possible by selection of the appropriate button. Result views are more condensed and provide more intuitive data access. This is particularly helpful when viewing properties of well-characterized enzymes for which BRENDA often includes thousands of parameters in all categories and an extended bibliography. Literature references and enzyme-associated diseases are now presented in separate windows.

### Conclusions and outlook

In addition to a substantial increase of data BRENDA has received major improvements in functionality. The associated modules for the EnzymeDetector and BKM-react have been enhanced. With the CAVEman human anatomy atlas human enzymes have received a new focus. Word maps and a new website afford an intuitive access. Presently the word maps are further improved to link them more closely to the BRENDA contents. A further user survey is in preparation to evaluate the functionality of the new website. Along with the next update of BRENDA at the end of 2014 the new version of BKM-react will include not only the BRENDA reactions classified as ‘natural’ but also all fully characterized BRENDA reactions including synthetic compounds. This will increase the number of unique reactions to more than 60 000.

## Availability

All described databases and features are accessible via the main BRENDA website: http://www.brenda-enzymes.org/. The EnzymeDetector can also be accessed via http://edbs.tu-bs.de/ and a direct link to BKM-react is provided via http://bkm-react.tu-bs.de/.
